# Supplementary figures and images for: Association of serum lysophosphatidylcholine acyltransferase 3 levels with metabolic variables and risk of type 2 diabetes mellitus: A cross-sectional study
Source: PLoS One. 2025 Jul 30;20(7):e0329301. doi: 10.1371/journal.pone.0329301 (PMC12310000; doi:10.1371/journal.pone.0329301)

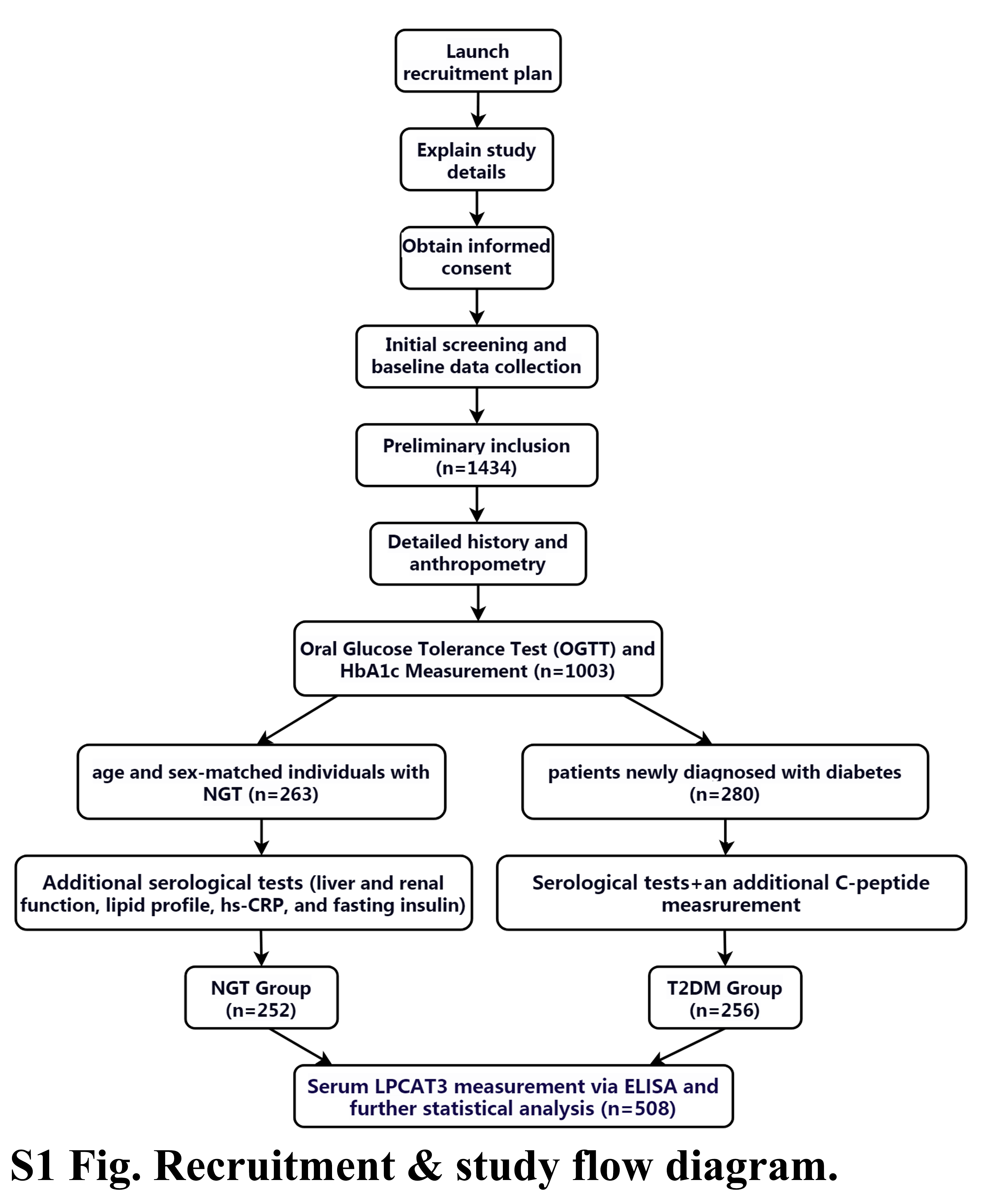

Supplement: S1 Fig — (TIF) [file pone.0329301.s001.tif]

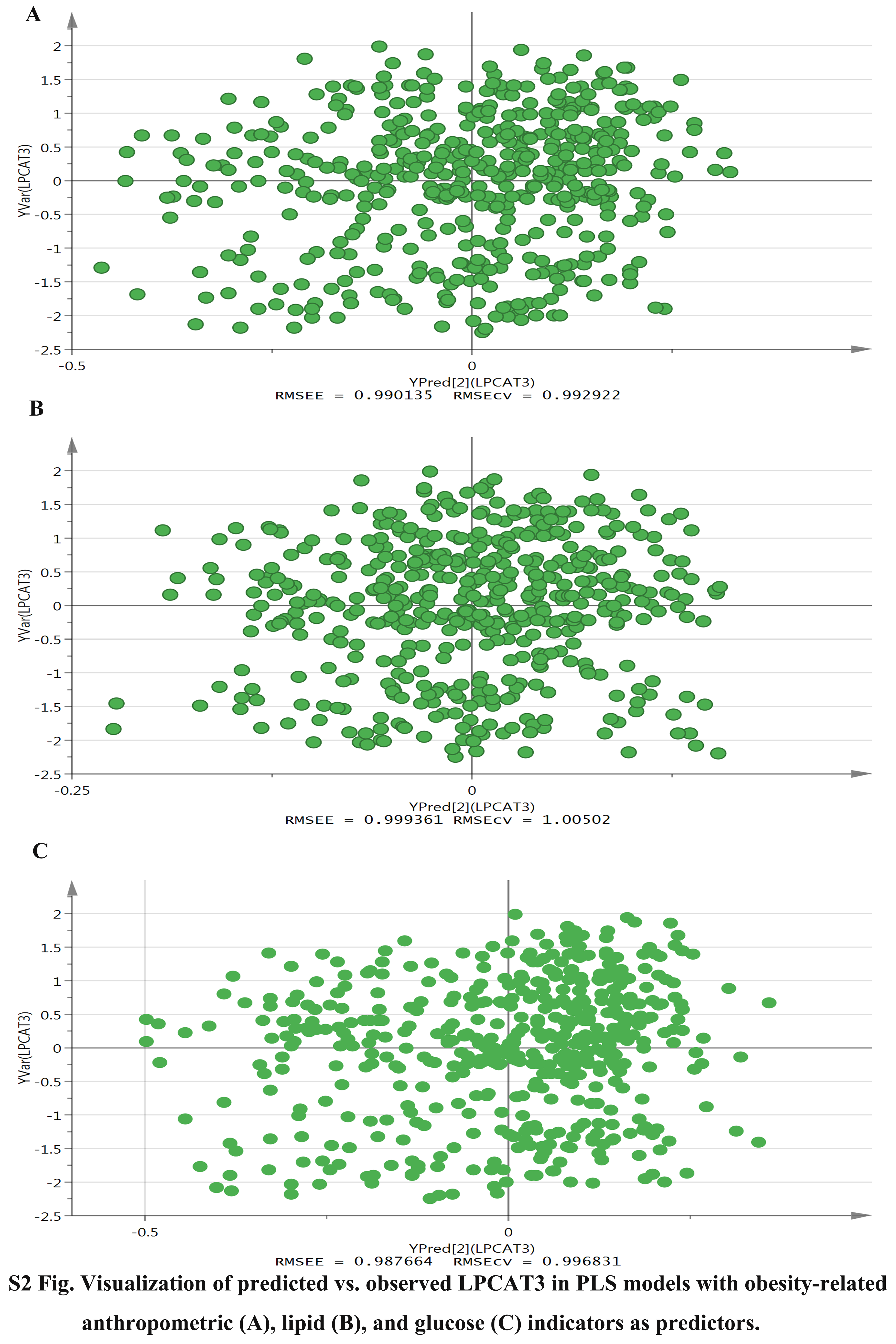

Supplement: S2 Fig — (TIF) [file pone.0329301.s002.tif]
