# Supplementary material for: Association of serum lysophosphatidylcholine acyltransferase 3 levels with metabolic variables and risk of type 2 diabetes mellitus: A cross-sectional study
Source: PLoS One. 2025 Jul 30;20(7):e0329301. doi: 10.1371/journal.pone.0329301 (PMC12310000; doi:10.1371/journal.pone.0329301)
Supplement: S1 Table — (DOCX) [file pone.0329301.s003.docx]

| **S1 Table. Comparison of serum LPCAT3 levels (ng/ml) based on liver and carotid status in NGT and T2DM populations.** | | |
| --- | --- | --- |
|  | **NGT** | **T2DM** |
| Non-fatty liver | 23.08 (14.17, 45.60) | 23.14 (11.27, 40.66) |
| Fatty liver | 22.91 (12.09, 48.16) | 20.75 (7.58, 33.52) |
| *z* | -0.684 | -1.131 |
| *p* | 0.494 | 0.258 |
| Non-carotid atherosclerosis | 23.28 (14.78, 47.70) | 21.87 (7.31, 35.45) |
| Carotid atherosclerosis | 22.42 (13.37, 63.85) | 23.14 (11.55, 39.99) |
| *z* | -0.301 | -1.298 |
| *p* | 0.763 | 0.194 |
| Data are presented as the median with the 25th percentile and 75th percentile, and inter-group comparisons are performed using the Mann-Whitney U test. Abbreviations: LPCAT3: lysophosphatidylcholine acyltransferase 3; NGT: normal glucose tolerance; T2DM: type 2 diabetes mellitus. | | |
