# Supplementary material for: Association of serum lysophosphatidylcholine acyltransferase 3 levels with metabolic variables and risk of type 2 diabetes mellitus: A cross-sectional study
Source: PLoS One. 2025 Jul 30;20(7):e0329301. doi: 10.1371/journal.pone.0329301 (PMC12310000; doi:10.1371/journal.pone.0329301)
Supplement: S2 Table — (DOCX) [file pone.0329301.s004.docx]

| **S2 Table. Evaluating the influence of confounding factors excluding obesity, lipid, and glucose parameters on a linear regression model for LPCAT3.** | | | | | | | |
| --- | --- | --- | --- | --- | --- | --- | --- |
| **Variables** | **unstandardised coefficients** | | ***t*** | ***p*** | **95% CI for *β*** | | **VIF** |
|  | ***β*** | **Std. Error** |  |  | **lower** | **upper** |  |
| Constant | 5.069 | 0.763 | 6.644 | <0.01 | 3.570 | 6.568 | - |
| BMI | -0.038 | 0.015 | -2.531 | <0.05 | -0.067 | -0.008 | 1.464 |
| HDL | -0.413 | 0.164 | -2.514 | <0.05 | -0.737 | -0.090 | 1.213 |
| FBG | -0.372 | 0.133 | -2.797 | <0.01 | -0.634 | -0.111 | 1.264 |
| Sex | 0.070 | 0.117 | 0.593 | 0.553 | -0.161 | 0.300 | 2.005 |
| Age | -0.003 | 0.004 | -0.785 | 0.433 | -0.011 | 0.005 | 1.197 |
| SBP | 0.000 | 0.004 | 0.120 | 0.905 | -0.007 | 0.007 | 1.780 |
| DBP | 8.820E-5 | 0.005 | 0.016 | 0.987 | -0.011 | 0.011 | 1.794 |
| ALT | -0.062 | 0.140 | -0.444 | 0.658 | -0.338 | 0.213 | 3.832 |
| AST | 0.025 | 0.211 | 0.119 | 0.905 | -0.389 | 0.439 | 3.329 |
| Cr | 0.002 | 0.004 | 0.414 | 0.679 | -0.006 | 0.009 | 1.965 |
| UA | 0.000 | 0.001 | 0.232 | 0.817 | -0.001 | 0.001 | 1.760 |
| hs-CRP | 0.001 | 0.043 | 0.025 | 0.980 | -0.084 | 0.086 | 1.202 |
| Incorporation of confounding factors (e.g., age, gender, blood pressure, liver/kidney function indicators) into the original linear model revealed their minimal impact on results, validating the model's continued applicability. The R Square of this model is 0.052. Prior to correlation analysis, LPCAT3, FBG, ALT, AST and hs-CRP were logarithmically transformed. Abbreviations: LPCAT3: lysophosphatidylcholine acyltransferase 3; CI: confidence interval; VIF: variance inflation factor; BMI: body mass index; HDL: high-density lipoprotein cholesterol; FBG: fasting blood glucose; SBP: systolic blood pressure; DBP: diastolic blood pressure; ALT: Alanine aminotransferase; AST: Aspartate aminotransferase; Cr: creatinine; UA: uric acid; hs-CRP: high sensitive C-reactive protein. | | | | | | | |
