# Supplementary material for: Association of serum lysophosphatidylcholine acyltransferase 3 levels with metabolic variables and risk of type 2 diabetes mellitus: A cross-sectional study
Source: PLoS One. 2025 Jul 30;20(7):e0329301. doi: 10.1371/journal.pone.0329301 (PMC12310000; doi:10.1371/journal.pone.0329301)
Supplement: S3 Table — (DOCX) [file pone.0329301.s005.docx]

| **S3 Table. Simple linear model considering only WHR as the independent variable.** | | | | | | | |
| --- | --- | --- | --- | --- | --- | --- | --- |
| **Variables** | **unstandardised coefficients** | | ***t*** | ***p*** | **95% CI for *β*** | | **VIF** |
|  | ***β*** | **Std. Error** |  |  | **lower** | **upper** |  |
| Constant | 4.815 | 0.678 | 7.100 | <0.01 | 3.482 | 6.147 | - |
| WHR | -1.990 | 0.740 | -2.690 | <0.01 | -3.443 | -0.537 | - |
| Including WHR alone in a simple regression model indicated a significant negative correlation between WHR and LPCAT3. The R Square of this model is 0.014. Prior to correlation analysis, LPCAT3 was logarithmically transformed. Abbreviations: LPCAT3: lysophosphatidylcholine acyltransferase 3; CI: confidence interval; VIF: variance inflation factor; WHR: waist-to-hip ratio. | | | | | | | |
