# Supplementary material for: Association of serum lysophosphatidylcholine acyltransferase 3 levels with metabolic variables and risk of type 2 diabetes mellitus: A cross-sectional study
Source: PLoS One. 2025 Jul 30;20(7):e0329301. doi: 10.1371/journal.pone.0329301 (PMC12310000; doi:10.1371/journal.pone.0329301)
Supplement: S4 Table — (DOCX) [file pone.0329301.s006.docx]

| **S4 Table. Incorporating WHR instead of BMI into the linear regression model.** | | | | | | | |
| --- | --- | --- | --- | --- | --- | --- | --- |
| **Variables** | **unstandardised coefficients** | | ***t*** | ***p*** | **95% CI for *β*** | | **VIF** |
|  | ***β*** | **Std. Error** |  |  | **lower** | **upper** |  |
| Constant | 5.301 | 0.732 | 7.242 | <0.01 | 3.862 | 6.739 | - |
| WHR | -1.337 | 0.795 | -1.681 | 0.093 | -2.899 | 0.225 | 1.180 |
| HDL | -0.310 | 0.151 | -2.053 | <0.05 | -0.607 | -0.013 | 1.028 |
| FBG | -0.391 | 0.127 | -3.072 | <0.01 | -0.641 | -0.141 | 1.161 |
| When WHR was incorporated instead of BMI into the multiple linear regression model, WHR did not show statistical significance. The R Square of this model is 0.039. Prior to correlation analysis, LPCAT3 and FBG were logarithmically transformed. Abbreviations: LPCAT3: lysophosphatidylcholine acyltransferase 3; CI: confidence interval; VIF: variance inflation factor; WHR: waist-to-hip ratio; HDL: high-density lipoprotein cholesterol; FBG: fasting blood glucose. | | | | | | | |
