# Supplementary material for: Association of serum lysophosphatidylcholine acyltransferase 3 levels with metabolic variables and risk of type 2 diabetes mellitus: A cross-sectional study
Source: PLoS One. 2025 Jul 30;20(7):e0329301. doi: 10.1371/journal.pone.0329301 (PMC12310000; doi:10.1371/journal.pone.0329301)
Supplement: S5 Table — (DOCX) [file pone.0329301.s007.docx]

| **S5 Table. Incorporating both BMI and WHR as independent variables into the linear regression model.** | | | | | | | |
| --- | --- | --- | --- | --- | --- | --- | --- |
| **Variables** | **unstandardised coefficients** | | ***t*** | ***p*** | **95% CI for *β*** | | **VIF** |
|  | ***β*** | **Std. Error** |  |  | **lower** | **upper** |  |
| Constant | 5.509 | 0.734 | 7.509 | <0.01 | 4.068 | 6.951 | - |
| BMI | -0.034 | 0.014 | -2.406 | <0.05 | -0.062 | -0.006 | 1.313 |
| WHR | -0.585 | 0.851 | -0.687 | 0.492 | -2.256 | 1.087 | 1.365 |
| HDL | -0.394 | 0.154 | -2.554 | <0.05 | -0.698 | -0.091 | 1.084 |
| FBG | -0.366 | 0.127 | -2.879 | <0.01 | -0.616 | -0.116 | 1.169 |
| When both BMI and WHR were incorporated as independent variables into the multiple linear regression model, BMI demonstrated statistical significance while WHR did not. The R Square of this model is 0.050. Prior to correlation analysis, LPCAT3 and FBG were logarithmically transformed. Abbreviations: LPCAT3: lysophosphatidylcholine acyltransferase 3; CI: confidence interval; VIF: variance inflation factor; BMI: body mass index; WHR: waist-to-hip ratio; HDL: high-density lipoprotein cholesterol; FBG: fasting blood glucose. | | | | | | | |
