# Supplementary material for: Association of serum lysophosphatidylcholine acyltransferase 3 levels with metabolic variables and risk of type 2 diabetes mellitus: A cross-sectional study
Source: PLoS One. 2025 Jul 30;20(7):e0329301. doi: 10.1371/journal.pone.0329301 (PMC12310000; doi:10.1371/journal.pone.0329301)
Supplement: S6 Table — (DOCX) [file pone.0329301.s008.docx]

| **S6 Table. Incorporating WC instead of BMI into the linear regression model.** | | | | | | | |
| --- | --- | --- | --- | --- | --- | --- | --- |
| **Variables** | **unstandardised coefficients** | | ***t*** | ***p*** | **95% CI for *β*** | | **VIF** |
|  | ***β*** | **Std. Error** |  |  | **lower** | **upper** |  |
| Constant | 5.315 | 0.518 | 10.264 | <0.01 | 4.298 | 6.333 | - |
| WC | -0.014 | 0.005 | -2.682 | <0.01 | -0.024 | -0.004 | 1.201 |
| HDL | -0.360 | 0.152 | -2.363 | <0.05 | -0.659 | -0.061 | 1.054 |
| FBG | -0.350 | 0.126 | -2.772 | <0.01 | -0.598 | -0.102 | 1.151 |
| When WC was substituted for BMI in the multiple linear regression model, a statistically significant model was still obtainable. The R Square of this model is 0.047. Prior to correlation analysis, LPCAT3 and FBG were logarithmically transformed. Abbreviations: LPCAT3: lysophosphatidylcholine acyltransferase 3; CI: confidence interval; VIF: variance inflation factor; WC: waist circumference; HDL: high-density lipoprotein cholesterol; FBG: fasting blood glucose. | | | | | | | |
