# Supplementary material for: Association of serum lysophosphatidylcholine acyltransferase 3 levels with metabolic variables and risk of type 2 diabetes mellitus: A cross-sectional study
Source: PLoS One. 2025 Jul 30;20(7):e0329301. doi: 10.1371/journal.pone.0329301 (PMC12310000; doi:10.1371/journal.pone.0329301)
Supplement: S7 Table — (DOCX) [file pone.0329301.s009.docx]

| **S7 Table. Incorporating both BMI and WHR as independent variables into the linear regression model.** | | | | | | | |
| --- | --- | --- | --- | --- | --- | --- | --- |
| **Variables** | **unstandardised coefficients** | | ***t*** | ***p*** | **95% CI for *β*** | | **VIF** |
|  | ***β*** | **Std. Error** |  |  | **lower** | **upper** |  |
| Constant | 5.289 | 0.518 | 10.208 | <0.01 | 4.271 | 6.307 | - |
| BMI | -0.026 | 0.022 | -1.190 | 0.235 | -0.069 | 0.017 | 3.119 |
| WC | -0.006 | 0.008 | -0.670 | 0.503 | -0.022 | 0.011 | 3.301 |
| HDL | -0.390 | 0.154 | -2.525 | <0.05 | -0.693 | -0.086 | 1.083 |
| FBG | -0.367 | 0.127 | -2.891 | <0.01 | -0.616 | -0.118 | 1.166 |
| When both BMI and WC were included as independent variables in the multiple linear regression model, despite no significant collinearity, neither variable showed statistical significance. The R Square of this model is 0.050. Prior to correlation analysis, LPCAT3 and FBG were logarithmically transformed. Abbreviations: LPCAT3: lysophosphatidylcholine acyltransferase 3; CI: confidence interval; VIF: variance inflation factor; BMI: body mass index; WC: waist circumference; HDL: high-density lipoprotein cholesterol; FBG: fasting blood glucose. | | | | | | | |
