# Supplementary material for: Association of serum lysophosphatidylcholine acyltransferase 3 levels with metabolic variables and risk of type 2 diabetes mellitus: A cross-sectional study
Source: PLoS One. 2025 Jul 30;20(7):e0329301. doi: 10.1371/journal.pone.0329301 (PMC12310000; doi:10.1371/journal.pone.0329301)
Supplement: S8 Table — (DOCX) [file pone.0329301.s010.docx]

| **S8 Table. Incorporating all lipid-related indicators into the regression model.** | | | | | | | |
| --- | --- | --- | --- | --- | --- | --- | --- |
| **Variables** | **unstandardised coefficients** | | ***t*** | ***p*** | **95% CI for *β*** | | **VIF** |
|  | ***β*** | **Std. Error** |  |  | **lower** | **upper** |  |
| Constant | 5.240 | 0.497 | 10.598 | <0.01 | 4.263 | 6.217 | - |
| BMI | -0.041 | 0.014 | -3.000 | <0.01 | -0.067 | -0.014 | 1.215 |
| HDL | -0.415 | 0.218 | -1.948 | 0.052 | -0.842 | 0.012 | 2.051 |
| FBG | -0.416 | 0.124 | -3.367 | <0.01 | -0.659 | -0.173 | 1.108 |
| TG | 0.039 | 0.089 | 0.456 | 0.648 | -0.136 | 0.213 | 1.792 |
| PC(TC&LDL) | 0.028 | 0.057 | 0.512 | 0.609 | -0.084 | 0.140 | 1.809 |
| When all lipid-related indicators were incorporated into the multiple liner regression model, HDL lost its statistical significance. The R Square of this model is 0.051. Prior to correlation analysis, LPCAT3, FBG and TG were logarithmically transformed. Abbreviations: LPCAT3: lysophosphatidylcholine acyltransferase 3; CI: confidence interval; VIF: variance inflation factor; BMI: body mass index; HDL: high-density lipoprotein cholesterol; FBG: fasting blood glucose; TG: triglyceride; PC(TC&LDL) denotes the principal component analysis of total cholesterol (TC) and low-density lipoprotein cholesterol (LDL). | | | | | | | |
