# Supplementary material for: Association of serum lysophosphatidylcholine acyltransferase 3 levels with metabolic variables and risk of type 2 diabetes mellitus: A cross-sectional study
Source: PLoS One. 2025 Jul 30;20(7):e0329301. doi: 10.1371/journal.pone.0329301 (PMC12310000; doi:10.1371/journal.pone.0329301)
Supplement: S9 Table — (DOCX) [file pone.0329301.s011.docx]

| **S9 Table. Incorporating 2hPG instead of FBG into the linear regression model.** | | | | | | | |
| --- | --- | --- | --- | --- | --- | --- | --- |
| **Variables** | **unstandardised coefficients** | | ***t*** | ***p*** | **95% CI for *β*** | | **VIF** |
|  | ***β*** | **Std. Error** |  |  | **lower** | **upper** |  |
| Constant | 5.085 | 0.449 | 11.320 | <0.01 | 4.203 | 5.968 | - |
| BMI | -0.039 | 0.013 | -2.932 | <0.01 | -0.065 | -0.013 | 1.140 |
| HDL | -0.415 | 0.155 | -2.674 | <0.01 | -0.721 | -0.110 | 1.093 |
| 2hPG | -0.290 | 0.107 | -2.718 | <0.01 | -0.499 | -0.080 | 1.085 |
| When 2hPG was substituted for FBG in the multiple linear regression model, a statistically significant model was still obtainable. The R Square of this model is 0.043. Prior to correlation analysis, LPCAT3 and 2hPG were logarithmically transformed. Abbreviations: LPCAT3: lysophosphatidylcholine acyltransferase 3; CI: confidence interval; VIF: variance inflation factor; WC: waist circumference; HDL: high-density lipoprotein cholesterol; 2hPG: 2-hour post-oral glucose tolerance test blood glucose level. | | | | | | | |
