# Supplementary material for: Association of serum lysophosphatidylcholine acyltransferase 3 levels with metabolic variables and risk of type 2 diabetes mellitus: A cross-sectional study
Source: PLoS One. 2025 Jul 30;20(7):e0329301. doi: 10.1371/journal.pone.0329301 (PMC12310000; doi:10.1371/journal.pone.0329301)
Supplement: S11 Table — (DOCX) [file pone.0329301.s013.docx]

| **S11 Table. Incorporating HOMA-IR instead of FBG into the linear regression model.** | | | | | | | |
| --- | --- | --- | --- | --- | --- | --- | --- |
| **Variables** | **unstandardised coefficients** | | ***t*** | ***p*** | **95% CI for *β*** | | **VIF** |
|  | ***β*** | **Std. Error** |  |  | **lower** | **upper** |  |
| Constant | 4.481 | 0.418 | 10.760 | <0.01 | 4.481 | .418 | - |
| BMI | -0.036 | 0.014 | -3.007 | <0.01 | -0.036 | 0.014 | 1.131 |
| HDL | -0.394 | 0.155 | -2.629 | <0.01 | -0.394 | 0.155 | 1.090 |
| HOMA-IR | -0.129 | 0.064 | -2.666 | <0.05 | -0.129 | 0.064 | 1.069 |
| When HOMA-IR was substituted for FBG in the multiple linear regression model, a statistically significant model was still obtainable. The R Square of this model is 0.043. Prior to correlation analysis, LPCAT3 and HOMA-IR were logarithmically transformed. Abbreviations: LPCAT3: lysophosphatidylcholine acyltransferase 3; CI: confidence interval; VIF: variance inflation factor; WC: waist circumference; HDL: high-density lipoprotein cholesterol; HOMA-IR: homeostasis model assessment of insulin resistance. | | | | | | | |
