# Supplementary material for: Association of serum lysophosphatidylcholine acyltransferase 3 levels with metabolic variables and risk of type 2 diabetes mellitus: A cross-sectional study
Source: PLoS One. 2025 Jul 30;20(7):e0329301. doi: 10.1371/journal.pone.0329301 (PMC12310000; doi:10.1371/journal.pone.0329301)
Supplement: S12 Table — (DOCX) [file pone.0329301.s014.docx]

| **S12 Table. Incorporating both FBG and 2hPG as independent variables into the linear regression model.** | | | | | | | |
| --- | --- | --- | --- | --- | --- | --- | --- |
| **Variables** | **unstandardised coefficients** | | ***t*** | ***p*** | **95% CI for *β*** | | **VIF** |
|  | ***β*** | **Std. Error** |  |  | **lower** | **upper** |  |
| Constant | 5.146 | 0.449 | 11.449 | <0.01 | 4.263 | 6.029 | - |
| BMI | -0.037 | 0.013 | -2.802 | <0.01 | -0.063 | -0.011 | 1.146 |
| HDL | -0.398 | 0.155 | -2.562 | <0.05 | -0.703 | -0.093 | 1.097 |
| FBG | -0.332 | 0.184 | -1.811 | 0.071 | -0.693 | 0.028 | 2.436 |
| 2hPG | -0.070 | 0.161 | -0.437 | 0.662 | -0.387 | 0.246 | 2.493 |
| When both FBG and 2hPG were included as independent variables in the multiple linear regression model, neither variable showed statistical significance. The R Square of this model is 0.050. Prior to correlation analysis, LPCAT3 and FBG were logarithmically transformed. Abbreviations: LPCAT3: lysophosphatidylcholine acyltransferase 3; CI: confidence interval; VIF: variance inflation factor; BMI: body mass index; HDL: high-density lipoprotein cholesterol; FBG: fasting blood glucose; 2hPG: 2-hour post-oral glucose tolerance test blood glucose level. | | | | | | | |
