# Supplementary material for: Association of serum lysophosphatidylcholine acyltransferase 3 levels with metabolic variables and risk of type 2 diabetes mellitus: A cross-sectional study
Source: PLoS One. 2025 Jul 30;20(7):e0329301. doi: 10.1371/journal.pone.0329301 (PMC12310000; doi:10.1371/journal.pone.0329301)
Supplement: S13 Table — (DOCX) [file pone.0329301.s015.docx]

| **S13 Table. Incorporating both FBG and HbA1c as independent variables into the linear regression model.** | | | | | | | |
| --- | --- | --- | --- | --- | --- | --- | --- |
| **Variables** | **unstandardised coefficients** | | ***t*** | ***p*** | **95% CI for *β*** | | **VIF** |
|  | ***β*** | **Std. Error** |  |  | **lower** | **upper** |  |
| Constant | 5.044 | 0.519 | 9.715 | <0.01 | 4.024 | 6.064 | - |
| BMI | -0.038 | 0.013 | -2.863 | <0.01 | -0.063 | -0.012 | 1.137 |
| HDL | -0.386 | 0.155 | -2.489 | <0.05 | -0.691 | -0.081 | 1.096 |
| FBG | -0.438 | 0.237 | -1.848 | 0.065 | -0.904 | 0.028 | 4.068 |
| HbA1c | 0.075 | 0.336 | 0.224 | 0.823 | -0.585 | 0.735 | 4.100 |
| When both FBG and HbA1c were included as independent variables in the multiple linear regression model, neither variable showed statistical significance. The R Square of this model is 0.049. Prior to correlation analysis, LPCAT3 and FBG were logarithmically transformed. Abbreviations: LPCAT3: lysophosphatidylcholine acyltransferase 3; CI: confidence interval; VIF: variance inflation factor; BMI: body mass index; HDL: high-density lipoprotein cholesterol; FBG: fasting blood glucose; HbA1c: glycated hemoglobin A1c. | | | | | | | |
