# Supplementary material for: Association of serum lysophosphatidylcholine acyltransferase 3 levels with metabolic variables and risk of type 2 diabetes mellitus: A cross-sectional study
Source: PLoS One. 2025 Jul 30;20(7):e0329301. doi: 10.1371/journal.pone.0329301 (PMC12310000; doi:10.1371/journal.pone.0329301)
Supplement: S14 Table — (DOCX) [file pone.0329301.s016.docx]

| **S14 Table. Incorporating all glucose-related indicators into the regression model.** | | | | | | | |
| --- | --- | --- | --- | --- | --- | --- | --- |
| **Variables** | **unstandardised coefficients** | | ***t*** | ***p*** | **95% CI for *β*** | | **VIF** |
|  | ***β*** | **Std. Error** |  |  | **lower** | **upper** |  |
| Constant | 4.936 | 0.569 | 8.681 | <0.01 | 3.819 | 6.053 |  |
| BMI | -0.036 | 0.014 | -2.602 | <0.01 | -0.064 | -0.009 | 1.278 |
| HDL | -0.394 | 0.156 | -2.526 | <0.05 | -0.701 | -0.088 | 1.103 |
| FBG | -0.425 | 0.253 | -1.680 | 0.094 | -0.923 | 0.072 | 4.624 |
| 2hPG | -0.164 | 0.213 | -0.769 | 0.442 | -0.582 | 0.255 | 4.340 |
| HbA1c | 0.299 | 0.444 | 0.673 | 0.502 | -0.574 | 1.171 | 7.140 |
| HOMA-IR | -0.007 | 0.080 | -0.091 | 0.927 | -0.165 | 0.150 | 1.899 |
| When all glucose-related indicators were incorporated into the multiple regression model, some, but not highly significant, collinearity was observed among them, and none of the blood glucose indicators demonstrated statistical significance. The R Square of this model is 0.050. Prior to correlation analysis, LPCAT3, FBG, 2hPG, HbA1c and HOMA-IR were logarithmically transformed. Abbreviations: LPCAT3: lysophosphatidylcholine acyltransferase 3; CI: confidence interval; VIF: variance inflation factor; BMI: body mass index; HDL: high-density lipoprotein cholesterol; FBG: fasting blood glucose; 2hPG: 2-hour post-oral glucose tolerance test blood glucose level; HbA1c: glycated hemoglobin A1c; HOMA-IR: homeostasis model assessment of insulin resistance. | | | | | | | |
