# Supplementary material for: Association of serum lysophosphatidylcholine acyltransferase 3 levels with metabolic variables and risk of type 2 diabetes mellitus: A cross-sectional study
Source: PLoS One. 2025 Jul 30;20(7):e0329301. doi: 10.1371/journal.pone.0329301 (PMC12310000; doi:10.1371/journal.pone.0329301)
Supplement: S15 Table — (DOCX) [file pone.0329301.s017.docx]

| **S15 Table. Selected cut-off points based on serum LPCAT3 levels for ROC analysis of T2DM prediction.** | | | | |
| --- | --- | --- | --- | --- |
| Cut-off Point | Sensitivity (95% CI) | Specificity (95% CI) | +LR | -LR |
| **≥2.387** | 100 (98.6 - 100.0) | 0 (0.0 - 1.5) | 1 | - |
| **>3.302** | 98.83 (96.6 - 99.8) | 7.14 (4.3 - 11.1) | 1.06 | 0.16 |
| **>5.028** | 92.58 (88.7 - 95.5) | 15.87 (11.6 - 21.0) | 1.1 | 0.47 |
| **>7.914** | 82.03 (76.8 - 86.5) | 23.81 (18.7 - 29.6) | 1.08 | 0.75 |
| **>11.003** | 79.3 (73.8 - 84.1) | 29.37 (23.8 - 35.4) | 1.12 | 0.71 |
| **>15.025** | 74.61 (68.8 - 79.8) | 34.52 (28.7 - 40.7) | 1.14 | 0.74 |
| **>20.118** | 57.81 (51.5 - 63.9) | 46.83 (40.5 - 53.2) | 1.09 | 0.9 |
| **>25.242** | 48.83 (42.6 - 55.1) | 60.71 (54.4 - 66.8) | 1.24 | 0.84 |
| **>30.112** | **42.19 (36.1 - 48.5)** | **71.83 (65.8 - 77.3)** | **1.5** | **0.8** |
| **>35.222** | 36.72 (30.8 - 42.9) | 75 (69.2 - 80.2) | 1.47 | 0.84 |
| **>40.448** | 28.91 (23.4 - 34.9) | 81.75 (76.4 - 86.3) | 1.58 | 0.87 |
| **>45.228** | 27.34 (22.0 - 33.2) | 85.71 (80.8 - 89.8) | 1.91 | 0.85 |
| **>50.2** | 23.83 (18.7 - 29.5) | 87.3 (82.5 - 91.1) | 1.88 | 0.87 |
| **>60.228** | 15.23 (11.1 - 20.2) | 92.06 (88.0 - 95.1) | 1.92 | 0.92 |
| **>70.317** | 13.28 (9.4 - 18.1) | 96.03 (92.8 - 98.1) | 3.35 | 0.9 |
| **>104.388** | 1.95 (0.6 - 4.5) | 99.6 (97.8 - 100.0) | 4.92 | 0.98 |
| This table presents the outcomes of an ROC curve analysis aimed at predicting the incidence of Type 2 Diabetes Mellitus (T2DM) based on serum LPCAT3 levels. It lists selected cut-off points along with their corresponding sensitivity, specificity, positive likelihood ratio (+LR), and negative likelihood ratio (-LR). Specifically, the cut-off point ">30.112" strikes a notable balance between sensitivity (42.19%, 95% CI: 36.1 - 48.5) and specificity (71.83%, 95% CI: 65.8 - 77.3). | | | | |
