# Supplementary material for: Association of serum lysophosphatidylcholine acyltransferase 3 levels with metabolic variables and risk of type 2 diabetes mellitus: A cross-sectional study
Source: PLoS One. 2025 Jul 30;20(7):e0329301. doi: 10.1371/journal.pone.0329301 (PMC12310000; doi:10.1371/journal.pone.0329301)
Supplement: S16 Table — (DOCX) [file pone.0329301.s018.docx]

| **S16 Table. Gender-stratified regression analysis of the association between LPCAT3 and metabolic parameters (BMI, HDL, FBG).** | | | | | | | |
| --- | --- | --- | --- | --- | --- | --- | --- |
| **gender** | **variables** | **unstandardised coefficients** | | ***t*** | ***p*** | **95% CI for *β*** | |
|  |  | ***β*** | **Std. Error** |  |  | **lower** | **upper** |
| male (n=259) | Constant | 5.625 | 0.608 | 9.253 | <0.01 | 4.428 | 6.823 |
|  | BMI | -0.035 | 0.018 | -1.979 | <0.05 | -0.069 | -0.000 |
|  | HDL | -0.568 | 0.226 | -2.516 | <0.05 | -1.012 | -0.123 |
|  | FBG | -0.613 | 0.158 | -3.888 | <0.01 | -0.924 | -0.303 |
| female (n=249) | Constant | 4.621 | 0.636 | 7.270 | <0.01 | 3.369 | 5.873 |
|  | BMI | -0.047 | 0.020 | -2.328 | <0.05 | -0.087 | -0.007 |
|  | HDL | -0.251 | 0.222 | -1.128 | 0.261 | -0.688 | 0.187 |
|  | FBG | -0.083 | 0.190 | -0.435 | 0.664 | -0.458 | 0.292 |
| The results are presented as coefficients, t-values, p-values, and 95% confidence intervals (CIs). A p-value < 0.05 was considered statistically significant, indicating a significant relationship between the corresponding variable and LPCAT3. The male model had an R-Square of 0.089, while the female model had an R-Square of 0.029. Prior to correlation analysis, LPCAT3 and FBG were logarithmically transformed. Abbreviations: LPCAT3, lysophosphatidylcholine acyltransferase 3; BMI, body mass index; HDL, high-density lipoprotein cholesterol; FBG, fasting blood glucose. | | | | | | | |
