# Supplementary material for: Association of serum lysophosphatidylcholine acyltransferase 3 levels with metabolic variables and risk of type 2 diabetes mellitus: A cross-sectional study
Source: PLoS One. 2025 Jul 30;20(7):e0329301. doi: 10.1371/journal.pone.0329301 (PMC12310000; doi:10.1371/journal.pone.0329301)
Supplement: S17 Table — (DOCX) [file pone.0329301.s019.docx]

| **S17 Table. Age-stratified regression analysis of the association between LPCAT3 and metabolic parameters (BMI, HDL, FBG).** | | | | | | | |
| --- | --- | --- | --- | --- | --- | --- | --- |
| **age groups (years)** | **variables** | **unstandardised coefficients** | | ***t*** | ***p*** | **95% CI for *β*** | |
|  |  | ***β*** | **Std. Error** |  |  | **lower** | **upper** |
| <40 (n=71) | Constant | 4.758 | 1.038 | 4.582 | <0.01 | 2.685 | 6.831 |
|  | BMI | -0.030 | 0.029 | -1.041 | 0.302 | -0.089 | 0.028 |
|  | HDL | -0.365 | 0.398 | -0.918 | 0.362 | -1.159 | 0.429 |
|  | FBG | -0.313 | 0.245 | -1.275 | 0.207 | -0.802 | 0.177 |
| 40-59 (n=315) | Constant | 4.799 | 0.581 | 8.262 | <0.01 | 3.656 | 5.942 |
|  | BMI | -0.026 | 0.017 | -1.490 | 0.137 | -0.059 | 0.008 |
|  | HDL | -0.329 | 0.197 | -1.666 | 0.097 | -0.717 | 0.060 |
|  | FBG | -0.419 | 0.157 | -2.669 | <0.01 | -0.727 | -0.110 |
| ≥60 (n=122) | Constant | 6.295 | 0.949 | 6.635 | <0.01 | 4.416 | 8.175 |
|  | BMI | -0.082 | 0.030 | -2.711 | <0.01 | -0.142 | -0.022 |
|  | HDL | -0.480 | 0.326 | -1.474 | 0.143 | -1.125 | 0.165 |
|  | FBG | -0.428 | 0.304 | -1.404 | 0.163 | -1.030 | 0.175 |
| The results are presented as coefficients, t-values, p-values, and 95% confidence intervals (CIs). A p-value less than 0.05 was considered statistically significant, indicating a significant relationship between the corresponding variable and LPCAT3. The models for the age groups <40, 40-59, and ≥60 had R-Square values of 0.052, 0.038, and 0.097, respectively. Prior to the correlation analysis, LPCAT3 and FBG were logarithmically transformed. Abbreviations: LPCAT3, lysophosphatidylcholine acyltransferase 3; BMI, body mass index; HDL, high-density lipoprotein cholesterol; FBG, fasting blood glucose. | | | | | | | |
