# Supplementary material for: Association of serum lysophosphatidylcholine acyltransferase 3 levels with metabolic variables and risk of type 2 diabetes mellitus: A cross-sectional study
Source: PLoS One. 2025 Jul 30;20(7):e0329301. doi: 10.1371/journal.pone.0329301 (PMC12310000; doi:10.1371/journal.pone.0329301)
Supplement: S18 Table — (DOCX) [file pone.0329301.s020.docx]

| **S18 Table. Logistic regression analysis of T2DM risk, with serum LPCAT3 as an independent predictor.** | | | | | |
| --- | --- | --- | --- | --- | --- |
| **Variables** | **unstandardised coefficients** | | Wald χ² | ***p*** | **Exp(*β*) (95% CI)** |
|  | ***β*** | **Std. Error** |  |  |  |
| Constant | 0.954 | 0.302 | 9.969 | <0.01 | 2.597 |
| LPCAT3 | -0.324 | 0.096 | 11.331 | <0.01 | 0.723 (0.599, 0.873) |
| The results are presented as coefficients (β), standard errors, Wald χ² values, p - values, odds ratios (OR), and 95% CI for the odds ratios. A p-value less than 0.05 was considered statistically significant, indicating a significant relationship between the corresponding variable and T2DM occurrence. The OR represents the multiplicative change in the odds of T2DM occurrence for a one-unit increase in the logarithmically transformed (using natural logarithm) serum LPCAT3 levels. In this case, an OR of 0.723 suggests that for every one-unit increase in the logarithmically transformed LPCAT3 levels, the odds of T2DM occurrence decrease by about 27.7% (since 1 - 0.723 = 0.277). Prior to analysis, serum LPCAT3 levels were logarithmically transformed using the natural logarithm (base e). Abbreviations: LPCAT3, lysophosphatidylcholine acyltransferase 3; CI, confidence interval; T2DM, type 2 diabetes mellitus. | | | | | |
