# Supplementary material for: Association of serum lysophosphatidylcholine acyltransferase 3 levels with metabolic variables and risk of type 2 diabetes mellitus: A cross-sectional study
Source: PLoS One. 2025 Jul 30;20(7):e0329301. doi: 10.1371/journal.pone.0329301 (PMC12310000; doi:10.1371/journal.pone.0329301)
Supplement: S19 Table — (DOCX) [file pone.0329301.s021.docx]

| **S19 Table. Binary logistic regression assesses independent predictors of T2DM, including serum LPCAT3, demographic factors (sex, age, BMI), and their interactions.** | | | | | |
| --- | --- | --- | --- | --- | --- |
| **Variables** | **unstandardised coefficients** | | Wald χ² | ***p*** | **Exp(*β*) (95% CI)** |
|  | ***β*** | **Std. Error** |  |  |  |
| Constant | -3.284 | 3.061 | 1.151 | 0.283 | 0.037 |
| LPCAT3 | -0.257 | 0.967 | 0.071 | 0.790 | 0.773 (0.116, 5.146) |
| Sex | -0.831 | 0.629 | 1.746 | 0.186 | 0.435 (0.127, 1.495) |
| Age | -0.013 | 0.027 | 0.229 | 0.632 | 0.987 (0.936, 1.041) |
| BMI | 0.194 | 0.105 | 3.419 | 0.064 | 1.214 (0.988, 1.490) |
| LPCAT3*Sex | 0.281 | 0.200 | 1.971 | 0.160 | 1.325 (0.895, 1.961) |
| LPCAT3*Age | 0.009 | 0.009 | 0.990 | 0.320 | 1.009 (0.992, 1.026) |
| LPCAT3*BMI | -0.019 | 0.033 | 0.330 | 0.565 | 0.981 (0.920, 1.047) |
| Results are presented as coefficients (β), standard errors (SE), Wald χ² statistics, p-values, odds ratios (OR), and 95% confidence intervals (CIs) for ORs, with statistical significance defined at p < 0.05. In this analysis, no significant interactions were detected between log-transformed serum LPCAT3 levels and age, sex (male as reference), or BMI in relation to T2DM incidence. Notably, the inclusion of these interaction terms in the model attenuated the initially observed inverse association between LPCAT3 and T2DM risk, suggesting that while the main effect of LPCAT3 on T2DM risk may be modified by demographic or anthropometric factors. LPCAT3 levels were log-transformed prior to analysis. Abbreviations: LPCAT3, lysophosphatidylcholine acyltransferase 3; T2DM, type 2 diabetes mellitus; BMI, body mass index. | | | | | |
