# Supplementary material for: Association of serum lysophosphatidylcholine acyltransferase 3 levels with metabolic variables and risk of type 2 diabetes mellitus: A cross-sectional study
Source: PLoS One. 2025 Jul 30;20(7):e0329301. doi: 10.1371/journal.pone.0329301 (PMC12310000; doi:10.1371/journal.pone.0329301)
Supplement: S20 Table — (DOCX) [file pone.0329301.s022.docx]

| **S20 Table. Independent predictors of T2DM risk by gender identified by binary logistic regression, with serum LPCAT3 levels as a key predictor.** | | | | | | |
| --- | --- | --- | --- | --- | --- | --- |
| **gender** | **variables** | **unstandardised coefficients** | | Wald χ² | ***p*** | **Exp(*β*) (95% CI)** |
|  |  | ***β*** | **Std. Error** |  |  |  |
| male (n=259) | Constant | 1.434 | 0.435 | 10.868 | <0.01 | 4.194 |
|  | LPCAT3 | -0.467 | 0.138 | 11.358 | <0.01 | 0.627 (0.478, 0.823) |
| female (n=249) | Constant | 0.469 | 0.426 | 1.213 | 0.271 | 1.598 |
|  | LPCAT3 | -0.180 | 0.135 | 1.773 | 0.183 | 0.835 (0.641, 1.089) |
| Binary logistic regression analyses were conducted separately for males and females to identify independent predictors of T2DM occurrence. Serum LPCAT3 levels, logarithmically transformed using the natural logarithm (base e), were included as a key predictor. The results are presented as coefficients (β), standard errors, Wald χ² statistics, p-values, odds ratios (OR), and 95% confidence intervals for the odds ratios. A p-value less than 0.05 was considered statistically significant, indicating a significant association between the corresponding variable and T2DM risk. The odds ratio (OR) represents the multiplicative change in the odds of T2DM occurrence associated with a one-unit increase in the logarithmically transformed serum LPCAT3 levels. In males, an odds ratio of 0.627 suggests that for every one-unit increase in the logarithmically transformed LPCAT3 levels, the odds of T2DM occurrence decrease by approximately 37.3% (since 1 - 0.627 = 0.373). In females, the odds ratio of 0.835 suggests a smaller, non-significant decrease of approximately 16.5% (since 1 - 0.835 = 0.165). Abbreviations: LPCAT3, lysophosphatidylcholine acyltransferase 3; T2DM, type 2 diabetes mellitus. | | | | | | |
