# Supplementary material for: Association of serum lysophosphatidylcholine acyltransferase 3 levels with metabolic variables and risk of type 2 diabetes mellitus: A cross-sectional study
Source: PLoS One. 2025 Jul 30;20(7):e0329301. doi: 10.1371/journal.pone.0329301 (PMC12310000; doi:10.1371/journal.pone.0329301)
Supplement: S21 Table — (DOCX) [file pone.0329301.s023.docx]

| **S21 Table. Independent predictors of T2DM risk by age group identified by binary logistic regression, with serum LPCAT3 levels as a key predictor.** | | | | | | |
| --- | --- | --- | --- | --- | --- | --- |
| age groups (years) | **variables** | **unstandardised coefficients** | | Wald χ² | ***p*** | **Exp(*β*) (95% CI)** |
|  |  | ***β*** | **Std. Error** |  |  |  |
| <40 (n=71) | constant | 0.713 | 0.845 | 0.713 | 0.399 | 2.041 |
|  | LPCAT3 | -0.271 | 0.275 | 0.969 | 0.325 | 0.763 (0.444, 1.308) |
| 40-59 (n=315) | constant | 1.222 | 0.396 | 9.522 | <0.01 | 3.394 |
|  | LPCAT3 | -0.398 | 0.125 | 10.151 | <0.01 | 0.671 (0.525, 0.858) |
| ≥60 (n=122) | constant | 0.499 | 0.572 | 0.762 | 0.383 | 1.648 |
|  | LPCAT3 | -0.190 | 0.183 | 1.084 | 0.298 | 0.827 (0.578, 1.183) |
| Binary logistic regression analyses were conducted separately for different age groups to identify independent predictors of T2DM risk. Serum LPCAT3 levels, logarithmically transformed using the natural logarithm (base e), were included as a key predictor. The results are presented as coefficients (β), standard errors, Wald χ² statistics, p-values, odds ratios (OR), and 95% confidence intervals (CIs) for the odds ratios. A p-value less than 0.05 was considered statistically significant, indicating a significant relationship between the corresponding variable and T2DM occurrence. In the 40-59 age group, an odds ratio of 0.671 suggests that for every one-unit increase in the logarithmically transformed LPCAT3 levels, the odds of T2DM occurrence decrease by approximately 32.9% (since 1 - 0.671 = 0.329). In the <40 and ≥60 age groups, the odds ratios were not statistically significant (p > 0.05), indicating no significant relationship between LPCAT3 levels and T2DM occurrence in these age groups. Abbreviations: LPCAT3, lysophosphatidylcholine acyltransferase 3; T2DM, type 2 diabetes mellitus. | | | | | | |
