# Supplementary material for: Association of serum lysophosphatidylcholine acyltransferase 3 levels with metabolic variables and risk of type 2 diabetes mellitus: A cross-sectional study
Source: PLoS One. 2025 Jul 30;20(7):e0329301. doi: 10.1371/journal.pone.0329301 (PMC12310000; doi:10.1371/journal.pone.0329301)
Supplement: S22 Table — (DOCX) [file pone.0329301.s024.docx]

| **S24 Table. BMI-stratified regression analysis of the association between LPCAT3 and metabolic parameters (BMI, HDL, FBG).** | | | | | | | |
| --- | --- | --- | --- | --- | --- | --- | --- |
| **BMI** | **variables** | **unstandardised coefficients** | | ***t*** | ***p*** | **95% CI for *β*** | |
|  |  | ***β*** | **Std. Error** |  |  | **lower** | **upper** |
| <24 kg/m² (n=232) | Constant | 6.341 | 1.076 | 5.893 | <0.01 | 4.221 | 8.462 |
|  | BMI | -0.095 | 0.044 | -2.153 | <0.05 | -0.183 | -0.008 |
|  | HDL | -0.594 | 0.220 | -2.700 | <0.01 | -1.028 | -0.161 |
|  | FBG | -0.217 | 0.197 | -1.101 | 0.272 | -0.605 | 0.171 |
| ≥24 kg/m² (n=276) | Constant | 4.383 | 0.754 | 5.813 | <0.01 | 2.898 | 5.867 |
|  | BMI | -0.012 | 0.023 | -0.525 | 0.600 | -0.058 | 0.033 |
|  | HDL | -0.215 | 0.216 | -0.998 | 0.319 | -0.640 | 0.210 |
|  | FBG | -0.487 | 0.154 | -3.167 | <0.01 | -0.789 | -0.184 |
| The results are presented as coefficients, t-values, p-values, and 95% confidence intervals (CIs). A p-value < 0.05 was considered statistically significant, indicating a significant association between the corresponding variable and LPCAT3. The analysis was stratified by BMI categories (BMI < 24 kg/m² vs. BMI ≥ 24 kg/m²). Before conducting the regression analysis, LPCAT3 and FBG were logarithmically transformed. Abbreviations: LPCAT3, lysophosphatidylcholine acyltransferase 3; BMI, body mass index; HDL, high-density lipoprotein cholesterol; FBG, fasting blood glucose. | | | | | | | |
