# Supplementary material for: Association of serum lysophosphatidylcholine acyltransferase 3 levels with metabolic variables and risk of type 2 diabetes mellitus: A cross-sectional study
Source: PLoS One. 2025 Jul 30;20(7):e0329301. doi: 10.1371/journal.pone.0329301 (PMC12310000; doi:10.1371/journal.pone.0329301)
Supplement: S23 Table — (DOCX) [file pone.0329301.s025.docx]

| **S23 Table. Comparison of demographic and clinical parameters between male and female participants.** | | | | |
| --- | --- | --- | --- | --- |
| **variables** | **male** | **female** | ***z* or *t* value** | ***p* value** |
| N | 259 | 249 | - | - |
| Age (year) | 51.17 ± 12.28 | 52.89 ± 10.96 | -2.63 | <0.01 |
| BMI (kg/m2) | 25.33 ± 3.33 | 24.16 ± 3.27 | 4.03 | <0.01 |
| WC (cm) | 93.05 ± 9.08 | 88.95 ± 8.02 | 5.38 | <0.01 |
| WHR | 0.92 ± 0.06 | 0.91 ± 0.05 | 3.93 | <0.01 |
| SBP (mmHg) | 132.09 ± 14.57 | 129.45 ± 16.69 | 1.90 | 0.58 |
| DBP (mmHg) | 83.99 ± 10.27 | 79.67 ± 9.91 | 4.83 | <0.01 |
| ALT (U/L) | 24.00 (17.00, 34.00) | 17.00 (13.00, 26.00) | -5.72 | <0.01 |
| AST (U/L) | 21.00 (18.00, 26.00) | 19.00 (16.00, 24.00) | -3.42 | <0.01 |
| Cr (umol/L) | 70.19 ± 13.41 | 51.79 ± 9.80 | 17.60 | <0.01 |
| UA (umol/L) | 374.81 ± 85.75 | 285.94 ± 70.23 | 12.75 | <0.01 |
| eGFR (ml/min) | 104.34 ± 15.63 | 105.33 ± 13.54 | -0.76 | 0.45 |
| TC (mmol/L) | 4.68 ± 1.07 | 4.80 ± 1.07 | -1.21 | 0.23 |
| TG (mmol/L) | 1.55 (1.05, 2.69) | 1.28 (0.92, 1.93) | -3.89 | <0.01 |
| HDL (mmol/L) | 1.14 ± 0.26 | 1.29 ± 0.28 | -6.09 | <0.01 |
| LDL (mmol/L) | 3.01 ± 0.76 | 3.05 ± 0.79 | -0.67 | 0.50 |
| FBG (mmol/L) | 5.44 (4.80, 7.82) | 5.30 (4.82, 7.10) | -0.85 | 0.40 |
| 2hPG (mmol/L) | 8.25 (6.28, 12.34) | 7.44 (6.39, 11.90) | -0.49 | 0.62 |
| HbA1c (%) | 6.33 (5.48, 7.90) | 5.68 (5.47, 7.63) | -0.57 | 0.57 |
| HOMA-IR | 2.37 (1.50, 4.45) | 2.23 (1.40, 3.64) | -0.95 | 0.34 |
| hs-CRP (mg/L) | 0.84 (0.40, 1.62) | 0.73 (0.31, 1.54) | -1.73 | 0.08 |
| Continuous variables with a normal distribution are presented as mean ± standard deviation and compared between groups using the t-test. Variables not following a normal distribution are expressed as median (25th-75th percentiles) and compared using the Mann-Whitney U test. A p-value < 0.05 was considered statistically significant. Abbreviations: NGT, normal glucose tolerance; T2DM, type 2 diabetes mellitus; BMI, body mass index; WC, waist circumference; WHR, waist-to-hip ratio; SBP, systolic blood pressure; DBP, diastolic blood pressure; ALT, Alanine aminotransferase; AST, Aspartate aminotransferase; Cr, creatinine; UA, uric acid; eGFR, estimated glomerular filtration rate; TC, total cholesterol; TG, triglyceride; HDL, high-density lipoprotein cholesterol; LDL, low-density lipoprotein cholesterol; FBG, fasting blood glucose; 2hPG, 2-hour post-oral glucose tolerance test blood glucose level; HbA1c, glycated hemoglobin A1c; HOMA-IR, homeostasis model assessment of insulin resistance; hs-CRP, high-sensitive C-reactive protein. | | | | |
