# Supplementary material for: Association of serum lysophosphatidylcholine acyltransferase 3 levels with metabolic variables and risk of type 2 diabetes mellitus: A cross-sectional study
Source: PLoS One. 2025 Jul 30;20(7):e0329301. doi: 10.1371/journal.pone.0329301 (PMC12310000; doi:10.1371/journal.pone.0329301)
Supplement: S25 Table — (DOCX) [file pone.0329301.s027.docx]

| **S25 Table. Comparison of demographic and clinical characteristics among different age groups.** | | | | | |
| --- | --- | --- | --- | --- | --- |
| **variables** | **age <40 years** | **age 40-59 years** | **age ≥60 years** | ***X^2^* or H or F value** | **p-value** |
| N (male/female) | 71 (46/25) | 318 (158/157)^a^ | 122 (55/67)^a^ | 7.20 | <0.05 |
| BMI (kg/m2) | 26.06 ± 3.86 | 24.73 ± 3.27^a^ | 24.08 ± 3.01^a^ | 8.13 | <0.01 |
| WC (cm) | 93.97 ± 10.26 | 90.81 ± 8.73^a^ | 89.92 ± 7.77^a^ | 5.11 | <0.01 |
| WHR | 0.92 ± 0.06 | 0.92 ± 0.06 | 0.91 ± 0.05 | 1.63 | 0.20 |
| SBP (mmHg) | 127.57 ± 15.39 | 130.17 ± 16.16 | 134.29 ± 14.00^ab^ | 4.86 | <0.01 |
| DBP (mmHg) | 81.39 ± 10.55 | 81.92 ± 10.60 | 82.01 ± 9.46 | 0.09 | 0.91 |
| ALT (U/L) | 27.00 (13.00, 51.00) | 21.00 (15.00, 31.00) | 18.00 (13.00, 26.25)^ab^ | 11.40 | <0.01 |
| AST (U/L) | 21.00 (16.00, 29.00) | 20.00 (17.00, 25.00) | 20.50 (17.00, 25.00) | 0.22 | 0.90 |
| Cr (umol/L) | 61.61 ± 16.38 | 60.48 ± 14.38 | 62.70 ± 15.50 | 1.00 | 0.37 |
| UA (umol/L) | 383.27 ± 94.96 | 324.64 ± 87.93^a^ | 318.04 ± 83.17^a^ | 14.72 | <0.01 |
| eGFR (ml/min) | 122.30 ± 14.04 | 105.70 ± 11.15^a^ | 92.40 ± 11.17^ab^ | 151.48 | <0.01 |
| TC (mmol/L) | 4.96 ± 1.12 | 4.79 ± 1.06 | 4.49 ± 1.04^ab^ | 5.37 | <0.01 |
| TG (mmol/L) | 1.79 (1.05, 3.14) | 1.41 (1.00, 2.33)^a^ | 1.22 (0.91, 1.68)^ab^ | 17.06 | <0.01 |
| HDL (mmol/L) | 1.16 ± 0.28 | 1.22 ± 0.28 | 1.22 ± 0.27 | 1.68 | 0.19 |
| LDL (mmol/L) | 3.24 ± 0.84 | 3.06 ± 0.75 | 2.82 ± 0.77^ab^ | 7.53 | <0.01 |
| FBG (mmol/L) | 5.53 (4.66, 9.48) | 5.35 (4.84, 7.40) | 5.23 (4.73, 7.31) | 2.31 | 0.31 |
| 2hPG (mmol/L) | 7.31 (5.95, 12.67) | 7.94 (6.38, 12.01) | 7.47 (6.34, 12.27) | 0.23 | 0.89 |
| HbA1c (%) | 5.67 (5.39, 10.03) | 6.19 (5.51, 7.81) | 5.59 (5.46, 7.52) | 1.07 | 0.58 |
| HOMA-IR | 3.05 (1.76, 6.56) | 2.31 (1.53, 4.09)^a^ | 1.90 (1.31, 3.14)^ab^ | 14.81 | <0.01 |
| hs-CRP (mg/L) | 0.98 (0.47, 1.93) | 0.72 (0.36, 1.56)^a^ | 0.75 (0.34, 1.47)^a^ | 5.73 | 0.06 |
| Continuous variables following a normal distribution are expressed as mean ± standard deviation, and inter-group comparisons are conducted using One-Way ANOVA. For variables not following a normal distribution, they are presented as median (25th-75th percentiles), and inter-group comparisons are performed using the Kruskal-Wallis H test. Categorical variables are compared using the Chi-square test. A p-value of less than 0.05 is considered statistically significant. ^a^ vs age <40 years group, p <0.05; ^b^ vs age 40-59 years group, p <0.05. Abbreviations: NGT: normal glucose tolerance; T2DM: type 2 diabetes mellitus; BMI: body mass index; WC: waist circumference; WHR: waist-to-hip ratio; SBP: systolic blood pressure; DBP: diastolic blood pressure; ALT: Alanine aminotransferase; AST: Aspartate aminotransferase; Cr: creatinine; UA: uric acid; eGFR: estimated glomerular filtration rate; TC: total cholesterol; TG: triglyceride; HDL: high-density lipoprotein cholesterol; LDL: low-density lipoprotein cholesterol; FBG: fasting blood glucose; 2hPG: 2-hour post-oral glucose tolerance test blood glucose level; HbA1c: glycated hemoglobin A1c; HOMA-IR: homeostasis model assessment of insulin resistance; hs-CRP: high sensitive C-reactive protein. | | | | | |
