# Supplementary material for: Association of serum lysophosphatidylcholine acyltransferase 3 levels with metabolic variables and risk of type 2 diabetes mellitus: A cross-sectional study
Source: PLoS One. 2025 Jul 30;20(7):e0329301. doi: 10.1371/journal.pone.0329301 (PMC12310000; doi:10.1371/journal.pone.0329301)
Supplement: Attachment — Submitted filename: Response to Reviewers.docx [file pone.0329301.s029.docx]

Dear editors and reviewers,

We extend our heartfelt gratitude for the opportunity to revise our manuscript. We sincerely appreciate the insightful feedback and constructive criticism provided by the reviewers, whose expertise significantly enhanced the rigor and clarity of our work. Their suggestions—spanning experimental design, data analysis, and in-depth analytical scrutiny—were invaluable in refining our study.

**Response to Reviewer A’s Comments:**

1. We appreciate the reviewer's feedback on the keyword 'glycolipid'. To avoid confusion and more accurately reflect our study's focus, we have revised it to ‘lipid and glucose’.

2. We confirm that our study included adults aged 18 to 80 years who had not used any medication in the past 30 days to minimize potential confounding effects from medications on our metabolic outcome measures. We are fully aware that when recruiting potential participants, we encountered a relatively small number of eligible individuals aged 60 and above, despite this age group having the highest response rate. The stringent inclusion criteria, particularly the no-medication-use requirement, excluded many individuals who might otherwise have been interested in participating. This observation is valuable for future studies, as it highlights a potential limitation of our current approach. In future research, we will carefully consider the possibility of modifying the inclusion criteria. For instance, we might explore the feasibility of excluding only specific types of medications, such as antidiabetic drugs and other medications known to have a significant impact on the primary outcomes of interest. This adjustment could potentially increase the pool of eligible participants while still maintaining a reasonable level of control over confounding factors. Once again, we thank you for your constructive feedback, which has helped us to reflect on the strengths and limitations of our study design and has provided us with valuable insights for future research endeavors.

3. We have added the reference values for all biochemical parameters in Table 1, adhering to Chinese clinical standards.

4. We have included details on the serum LPCAT3 measurement method: Samples were diluted 1:4 with the kit buffer, and absorbance was measured at 450 nm using a standard curve ranging from 0 to 40 ng/ml.

5. Thank you very much for your constructive feedback. We've diligently revised the discussion section to emphasize our study's results and their comparison with existing literature.

6. We are extremely grateful for your thought-provoking suggestion to include prediabetic patients in our study. We recognize the importance of including prediabetic patients in our study to better understand LPCAT3's role in early glucose dysregulation. Although we identified a subgroup with impaired glucose tolerance (IGT) during OGTT screening. However, our study was originally conceived to focus on individuals with either normal glucose tolerance (NGT) or diabetes. This design was driven by our primary research questions, which necessitated clear-cut phenotypic differences to enable robust statistical comparisons. When planning the study, we gave priority to minimizing potential confounding factors linked to intermediate metabolic states, like prediabetes, to improve the interpretability of our results. Our aim was to sidestep selection bias and confidently attribute any observed differences in LPCAT3 levels to the distinct metabolic characteristics of the NGT and diabetic groups. While this strategy bolstered our primary analyses, we're fully aware that it restricted our capacity to thoroughly assess LPCAT3 across the entire spectrum of glucose metabolism. We whole-heartedly concur that future studies should incorporate prediabetic populations to fill this knowledge gap. Such research would offer invaluable insights into how LPCAT3 behaves during the transition from normal blood sugar levels to diabetes and its potential as a therapeutic target for early intervention. We sincerely thank you for pointing out this crucial avenue of research, and we'll make it a top priority in our future endeavors.

**Response to Reviewer B’s Comments:**

**The title:**

1. Thank you for your valuable suggestion. We have revised the title to more accurately reflect the study's cross-sectional design and objectives.

**The introduction:**

1. We have clarified the research gap regarding serum LPCAT3 and emphasized the importance of exploring its links with metabolic variables and T2DM risk.
2. We've clarified the primary (association between serum LPCAT3 levels and T2DM risk) and secondary outcomes (correlations with metabolic variables) at the end of the introduction as you suggested.

**In method:**

1. Thank you for emphasizing the need for clarity. This was a cross-sectional study conducted from July 1, 2024, to December 31, 2024. The inclusion and exclusion criteria are fully detailed in the Methods section, ensuring transparency and reproducibility.
2. We sincerely appreciate your attention to ethical compliance. Ethical approval was obtained before study initiation, and trial registration was completed. These details are now included in the Ethics Statement and Trial Registration sections.
3. This study adhered to the Declaration of Helsinki (2013 revision), ensuring participant rights and safety. A statement to this effect has been added to the Ethics Statement section.
4. We apologize for the oversight and thank you for the suggestion. We have included a CONSORT flow diagram (S1 Fig) in the supplementary material, detailing participant recruitment, screening, allocation, and follow-up. Due to the journal's possible figure-limit rules, we had to put it there. We hope for your understanding.

**In statistical analysis:**

1. Your suggestions regarding the control of collinearity and confounding factors are undoubtedly constructive. We consulted numerous senior researchers in the field, and all of them were astounded by your keen insight in this regard. They expressed concerns that our handling might not fully meet your high standards. Nonetheless, we are immensely grateful for the opportunity to have learned a great deal of professional knowledge from you through this process. We have made revisions to the Methods and Results sections based on your valuable feedback. We sincerely appreciate your guidance and the profound respect we hold for your expertise.
2. In response to your valuable suggestion, we have relocated the sample size estimation to the Methods section.

**In results:**

1. We've carefully explained all abbreviations that appear in the tables to ensure clarity.
2. Thank you for your guidance. We’ve now included detailed descriptions of data presentation methods, statistical tests used for comparisons, and the significance levels beneath each table and figure as suggested.
3. Thank you sincerely for your invaluable guidance on refining the ROC curve analysis. We’ve now incorporated the cutoff points, 95% confidence intervals, and a detailed table summarizing the area under the curve (AUC) results. Your expertise has been immensely educational, and we deeply appreciate the opportunity to improve our work under your supervision. If there are any remaining areas that require adjustment, please don’t hesitate to correct us—we hold your profound knowledge in the highest regard and are eager to ensure the manuscript meets your standards. Thank you again for your time and insight.

**Discussion:**

1. We sincerely appreciate your astute observation regarding the lack of discussion on the effects of age, sex, and comorbidities in our study. We fully recognize the importance of analyzing comorbidities. In response, we've added an analysis of liver and kidney function in the appropriate sections, as these are crucial comorbidity-related factors. During the study, we also collected liver and carotid artery ultrasound information. Although this wasn't part of our original research plan, many participants strongly requested these ultrasound examinations. So, we conducted them on a completely voluntary basis, informing the participants of their right to refuse to share the ultrasound results and obtaining their written consent. Initially, we decided not to include the ultrasound results in the manuscript because only a portion of the participants completed the examinations, and we didn't find any particularly significant findings at that time. It was a tough call to add these results during the review process, as we were concerned about potential negative consequences. However, we ultimately convinced all the authors to include them. This decision was not only a respectful and proactive response to your valuable suggestions but also driven by our realization that these results could be an important discovery, offering readers more insights and potentially benefiting future research. As for age and sex, we acknowledge that there has been relatively little research in this area in the past. To preserve the conciseness and flow of our manuscript, we opted not to delve into this aspect in detail. We sincerely appreciate your understanding of our decision, which was made with the aim of maintaining a clear and focused presentation of our findings. Once again, thank you for your insightful comments, which have undoubtedly helped us improve our study.

**Limitations and conclusion:**

1. We have revised our conclusion to acknowledge that while low serum LPCAT3 levels may be associated with an increased risk of T2DM, the underlying relationships are complex due to the interplay among glycemic indices, lipid profiles, and anthropometric measures. We acknowledge that our initial interpretation may have oversimplified these complexities, and we are grateful for your guidance in presenting them more accurately.
2. Regarding the study's limitations, we acknowledge the potential for referral bias due to our single-center, geographically limited sample, which may have led to an overrepresentation of certain patient groups. Additionally, we recognize the inadequacy in our control and analysis of confounding factors. For instance, we did not sufficiently account for variables such as diet, physical activity, body composition, blood lipid and pressure levels, organ function, and comorbidities. We sincerely regret this oversight and propose that future large-scale, diverse longitudinal studies utilize multivariate modeling to more comprehensively account for these factors. We sincerely apologize for any shortcomings in our study and are committed to learning from this experience. Your feedback will be invaluable in steering our future research efforts towards greater rigor and comprehensiveness.

We are immensely grateful for your invaluable insights. Given our current level of understanding, our revisions may still fall short in certain areas. We sincerely apologize for any oversights in our study and are committed to learning from this experience. We deeply appreciate the time and effort you have dedicated to reviewing our work.

Best regards,

Gaonian Zhao
