# Supplementary material for: Association of serum lysophosphatidylcholine acyltransferase 3 levels with metabolic variables and risk of type 2 diabetes mellitus: A cross-sectional study
Source: PLoS One. 2025 Jul 30;20(7):e0329301. doi: 10.1371/journal.pone.0329301 (PMC12310000; doi:10.1371/journal.pone.0329301)
Supplement: Attachment — Submitted filename: Response_to_Reviewers_auresp_2.docx [file pone.0329301.s030.docx]

Dear Dr. Hideto Sano and Reviewers,

We are truly grateful for the invaluable opportunity to revise our manuscript. We sincerely acknowledge that in the previous round, we handled some issues rather hastily. The professional and insightful comments from the reviewers have been extremely beneficial, allowing us to gain a deeper understanding of the shortcomings in our study. Due to our limited expertise, there may still be some inadequacies, and we deeply appreciate the time and effort you have invested in reviewing our work. We apologize for any inconvenience caused.

**First and foremost, we must openly admit that there was improper data handling in the previous version of the manuscript.** During the logarithmic conversion of non-normally distributed data, we adopted different bases, using 10 in some instances and the natural number e in others. This lack of consistency not only produced erratic results but also posed a significant hurdle for readers in discerning the relationships among the data. In this round of revisions, we have standardized all logarithmic conversions to use the natural number e as the base. We have clearly articulated this in the Methods section of the article and provided supplementary explanations in the footnotes of specific tables to aid readers in comprehending the results. Notably, certain coefficients in S2-14 have been revised. We have clearly marked these modifications and uploaded the modification records in a separate folder. We are acutely aware that this may cause substantial inconvenience during your review process. We sincerely apologize for our previous improper handling.

The following modifications have been made in response to the concerns raised by the reviewers.

**Major Points:**

1. We sincerely appreciate the reviewer's sharp insight into the confounding effect of obesity. We fully recognize that our prior way of expressing the related results was not proper. We have carefully reworked the relevant parts to make them more scientifically and rigorously presented.

2. We acknowledge the weak explanatory and predictive power of our models. We have explicitly stated these implications in the Discussion and Limitations sections. We acknowledge that, based on our data, the clinical utility of serum LPCAT3 as a predictive or explanatory biomarker appears to be minimal.

3. We recognize that the omission of age- and sex-specific analyses was a significant oversight. Age and sex are fundamental modifiers of metabolism, adiposity, and T2DM risk. We have now performed and presented analyses stratified by sex. We have tested whether the correlations between LPCAT3 and BMI/HDL/FBG hold for both men and women, and also tested for an interaction between LPCAT3 and sex/age on T2DM risk. These sections are located in the last paragraph of the Results section. We deeply regret our reckless oversight of this issue during the previous review round and are immensely grateful for your kindness in granting us another opportunity to revise. Given the differing views on how to address the issue, we consulted with several experts for their insights and, after much deliberation, finally reached a consensus. We are genuinely concerned about whether our handling is appropriate. If this has caused you any inconvenience during the review, we sincerely apologize.

**Section-Specific Responses**

**Abstract**

As mentioned above, we have revised the conclusion in the abstract to reflect the non-significance of the association after adjusting for obesity.

**Introduction**

We have added the reference (DOI: 10.18502/aacc.v10is2.17213) to the sentence "With the progress in metabolomics and lipidomics research, the critical role of lipid metabolism dysregulation in the onset and progression of T2DM has gained increasing recognition."

**Methods**

In the Limitations section, we've dissected the reasons for not considering pre-diabetes in the experimental design. We fully acknowledge that this design decision significantly impedes our holistic grasp of the glycemic spectrum.

**Results**

We have summarized the key takeaway from the complex collinearity analyses (S3-S14 Tables) in the main text of the results. This gives the reader a clearer sense of the data's complexity, specifically that the relationships are unstable and dependent on which correlated variable is included in the model.

We have briefly summarized the results of the PLS analysis and acknowledge that the observed correlations are weak and unreliable.

**Discussion**

During the revision process, we actively sought counsel from several experts. They unanimously conveyed their deep-seated respect for your professionalism and, notably, sang high praises for your modification suggestions regarding the Discussion section. We are profoundly grateful for your invaluable insights. Your statement “Focus on what your data clearly show and what they do not” was truly an eye-opener for us. Although we still harbor some uncertainty about the appropriateness of our revisions, we can distinctly perceive a substantial enhancement in the Discussion section, all thanks to your invaluable guidance. Your assistance is, without a doubt, an irreplaceable treasure for our research endeavor.

We explored the reasons why obesity attenuates the association between LPCAT3 and T2DM. Building on this, we hypothesized that the connection between LPCAT3 and T2DM is indirect, as it is influenced by factors like obesity, and we subsequently provided a brief discussion on this hypothesis.

We conducted a brief analysis on the potential differences in the origin and function of serum LPCAT3 compared to tissue-specific LPCAT3.

We have removed speculative phrasing that is not supported by our weak data.

The final manuscript was reviewed by two independent experts. They made some minor adjustments to the sentence expressions, aiming to minimize potential confusion for readers and enhance the fluency of the article. We acknowledge that these revisions may necessitate additional scrutiny during your re-examination. For any inconvenience this may cause, we extend our most sincere apologies, and express profound gratitude for your unwavering commitment to upholding the intellectual integrity of this scholarly endeavor.

The supervision of the revision process for this manuscript was undertaken by Professor Xingbo Cheng. He is a respected elder in the local community and has twice served as the chairman of the Jiangsu Diabetes Society. He specifically instructed us to convey to you his utmost respect for your outstanding professional knowledge and inclusive and magnanimous qualities.

During the revision process, we observed a marked improvement in the manuscript's quality. This valuable experience has not only deepened our knowledge but also laid a solid groundwork for our future research. Indeed, this submission has proven to be a highly constructive endeavor. We extend our heartfelt gratitude to the editors and all reviewers for their unwavering commitment to enhancing this manuscript. Your meticulous attention to detail and profoundly insightful suggestions have been of immense value to our work. However, acknowledging the limits of our expertise, we hesitate to assert that we've fully addressed all your concerns in the revision. We sincerely apologize for any inconvenience this may have caused and would greatly appreciate your continued guidance and support.

Sincerely,

Xingbo Cheng, Gaonian Zhao, Haifeng Zhu, et al. (on behalf of all the authors)

2025-07-01
